# Bilateral Erector Spinae Plane Block With 0.375% Ropivacaine and Postoperative Analgesia After Laparoscopic Cholecystectomy: A Prospective, Randomized, Controlled Cohort Study

**DOI:** 10.7759/cureus.117362

**Published:** 2026-10-02

**Authors:** Kenny Omar Contreras Becerra, Jesus Alberto Sanson-Riofrio, Juan Manuel Lopez Diaz, Lizethh Sanson Riofrio, Rodolfo Chávez Magallón, Angelica Morelia, Lilibeth Monroy Favela

**Affiliations:** 1 Department of Clinical Coordination for Education and Research/Anesthesiology, Hospital General de Zona No. 33, Instituto Mexicano del Seguro Social (IMSS) en Bahia de Banderas, Nayarit, MEX; 2 Department of Surgical Oncology, Hospital General de Zona No. 33, Instituto Mexicano del Seguro Social (IMSS) en Bahia de Banderas, Nayarit, MEX; 3 Department of Anesthesiology, Hospital General de Zona No. 33, Instituto Mexicano del Seguro Social (IMSS) en Bahia de Banderas, Nayarit, MEX; 4 Department of Dermatology, Fundacion Mexicana para la Dermatología A.C., Mexico City, MEX; 5 Department of General Surgery, Hospital General de Zona No. 33, Instituto Mexicano del Seguro Social (IMSS) en Bahia de Banderas, Nayarit, MEX; 6 Department of Epidemiology, Hospital General de Zona No. 33, Instituto Mexicano del Seguro Social (IMSS) en Bahia de Banderas, Nayarit, MEX

**Keywords:** erector spinae plane block, laparoscopic cholecystectomy, multimodal analgesia, postoperative analgesia, ropivacaine

## Abstract

Introduction: Postoperative pain after laparoscopic cholecystectomy may delay recovery and increase opioid exposure. The erector spinae plane block has been proposed as a component of multimodal analgesia, but local prospective data remain limited.

Methods: This prospective, randomized, controlled cohort study included 40 adults undergoing elective laparoscopic cholecystectomy. Of these, 20 participants received an ultrasound-guided bilateral erector spinae plane block at T9 with 0.375% ropivacaine (20 mL per side) plus multimodal analgesia, and the remaining 20 patients received multimodal analgesia without the block. The prespecified primary endpoint was resting Numeric Rating Scale pain at 12 hours. Secondary outcomes included pain at additional time points and recorded rescue tramadol use. Analyses were unadjusted and included repeated-measures models, Holm-adjusted pointwise comparisons, standardized mean differences, and Fisher exact tests.

Results: At 12 hours, mean resting pain was 1.80 ± 0.70 in the block group and 3.40 ± 1.31 in the comparison group (mean difference -1.60; 95% CI -2.28 to -0.92; p<0.001; Hedges' g=-1.49). The difference did not reach the prespecified two-point threshold used for sample size planning. Recorded rescue tramadol use occurred in two (10.0%) participants in the block group versus 14 (70.0%) participants in the comparison group (risk ratio 0.143; 95% CI 0.037-0.549; absolute risk difference -0.60; p=0.00024). The group-by-time interaction was not significant for resting or movement pain. Postoperative nausea and vomiting occurred in one participant per group.

Conclusions: Bilateral erector spinae plane block was associated with lower unadjusted pain scores and a marked reduction in recorded rescue tramadol use. The most clinically salient signal was the lower frequency of recorded rescue analgesia, although the indication for every administration could not be independently verified. Because the primary pain difference remained below the prespecified two-point threshold, the findings should be considered hypothesis-generating.

## Introduction

Laparoscopic cholecystectomy is commonly followed by a combination of somatic, visceral, and referred pain. Procedure-specific recommendations support multimodal analgesia to reduce opioid exposure and facilitate early recovery [1-3]. Despite the minimally invasive approach, a clinically relevant proportion of patients still require rescue opioids during the first postoperative day.

Ultrasound-guided fascial plane blocks have expanded the options for perioperative analgesia. The erector spinae plane block is intended to produce craniocaudal spread within a thoracic fascial plane and may influence both somatic and visceral nociception [4,5]. Systematic reviews of laparoscopic cholecystectomy have reported lower pain scores and opioid requirements with the block, although effect estimates vary across techniques, vertebral levels, local anesthetic regimens, and comparator strategies [6-8].

Prospective trials have generally supported an analgesic benefit, but most were randomized studies conducted outside Mexico [9-17]. The present study evaluated the association between bilateral erector spinae plane block with 0.375% ropivacaine and postoperative analgesic outcomes in a Mexican hospital. The prespecified primary endpoint was resting pain at 12 hours. Secondary outcomes included pain at other scheduled assessments, recorded rescue tramadol use, and crude postoperative nausea and vomiting.

## Materials and methods

Study design and setting

This was a prospective, randomized, controlled cohort study conducted at Hospital General de Zona No. 33 in Bahía de Banderas, Nayarit, Mexico, between January and May 2026. Participants were analyzed according to the perioperative analgesic strategy received. Reporting was structured according to the STROBE (STrengthening the Reporting of OBservational studies in Epidemiology) cohort-study framework [18].

Ethical considerations

The study protocol was approved on December 5, 2025, by the Local Health Research Committee No. 1802, General Hospital Zone No. 1, Instituto Mexicano del Seguro Social (IMSS) (registration number: R-2025-1802-061). Written informed consent was obtained from all participants before inclusion.

Study population

Eligible participants were adults aged 18-65 years with American Society of Anesthesiologists (ASA) physical status I-III who were scheduled for elective laparoscopic cholecystectomy under general anesthesia. Patients were excluded if they had a history of thoracic or abdominal neuralgia, psychiatric disorders, cognitive impairment, or declined participation. Patients requiring conversion from laparoscopic to open surgery or experiencing an anesthetic complication requiring protocol deviation were also excluded in the final analysis.

Exposure groups and perioperative analgesia

The analytic dataset contained 40 participants, with 20 participants in each exposure group. All participants received a standardized general anesthetic and intravenous multimodal analgesia comprising paracetamol 1 g, ketorolac 30 mg, dexamethasone 8 mg, and ondansetron 8 mg. Participants were assigned to Group A or Group B using a computer-generated allocation sequence created and used by an investigator who was not involved in clinical care or postoperative data collection. Group A (block group) received an ultrasound-guided bilateral erector spinae plane block at the T9 level before induction, using 0.375% ropivacaine at 20 mL per side (150 mg total). Group B (comparison group) received the same multimodal regimen without the block. Postoperative pain was assessed by a post-anesthesia care unit (PACU) physician who was not involved in the allocation process and did not have access to the allocation list or intraoperative block documentation. However, the original computer-generated allocation-sequence file, a separate record documenting allocation concealment, and a separate blinding log is no longer available and this has been listed in the Limitations.

Outcomes

Pain intensity was measured using an 11-point Numeric Rating Scale from 0 (no pain) to 10 (worst pain imaginable). Resting pain was assessed at 0, 2, 12, and 24 hours after surgery, and movement pain was assessed at 12 and 24 hours. The prespecified primary endpoint was resting pain at 12 hours. The secondary analgesic endpoint was recorded as rescue tramadol use. Intravenous tramadol 50 mg was intended for breakthrough pain at a Numeric Rating Scale score of at least 6; however, the contemporaneous clinical indication for every recorded administration could not be independently verified. Safety assessment was limited to the crude incidence of postoperative nausea and vomiting.

Sample size

The sample size was planned to detect a two-point difference in resting pain at 12 hours, assuming a standard deviation of 2.0, a two-sided alpha of 0.05, and 80% power. The final target was 20 participants per group after allowing for anticipated attrition. The two-point value is reported as the prespecified planning threshold rather than a universally established minimal clinically important difference.

Statistical analysis

Analyses were unadjusted because the sample size did not support stable multivariable modeling. Continuous baseline variables were compared with Welch t-tests and summarized with standardized mean differences. Pain trajectories were evaluated using mixed repeated-measures analysis of variance. Greenhouse-Geisser correction was applied when sphericity was violated, and partial eta squared quantified model-level effect size. Pointwise group comparisons used Welch t-tests with Holm correction; Hedges' g, a small-sample bias-corrected standardized mean difference, quantified the magnitude of between-group differences. Mann-Whitney U tests with Holm correction were used as sensitivity analyses. Categorical outcomes were compared using Fisher's exact test and summarized with risk ratios, absolute risk differences, and, for recorded rescue tramadol use, the number needed to treat. All effect estimates are crude associations, and two-sided p-values below 0.05 were considered statistically significant. Statistical analyses were performed using IBM SPSS Statistics for Windows, version 24.0 (IBM Corp., Armonk, New York, United States).

## Results

Participant flow and baseline characteristics

The analytic dataset included 40 complete records, with 20 participants in each group. No scheduled pain outcome was missing. Figure 1 presents the flow of participants.

**Figure 1:**
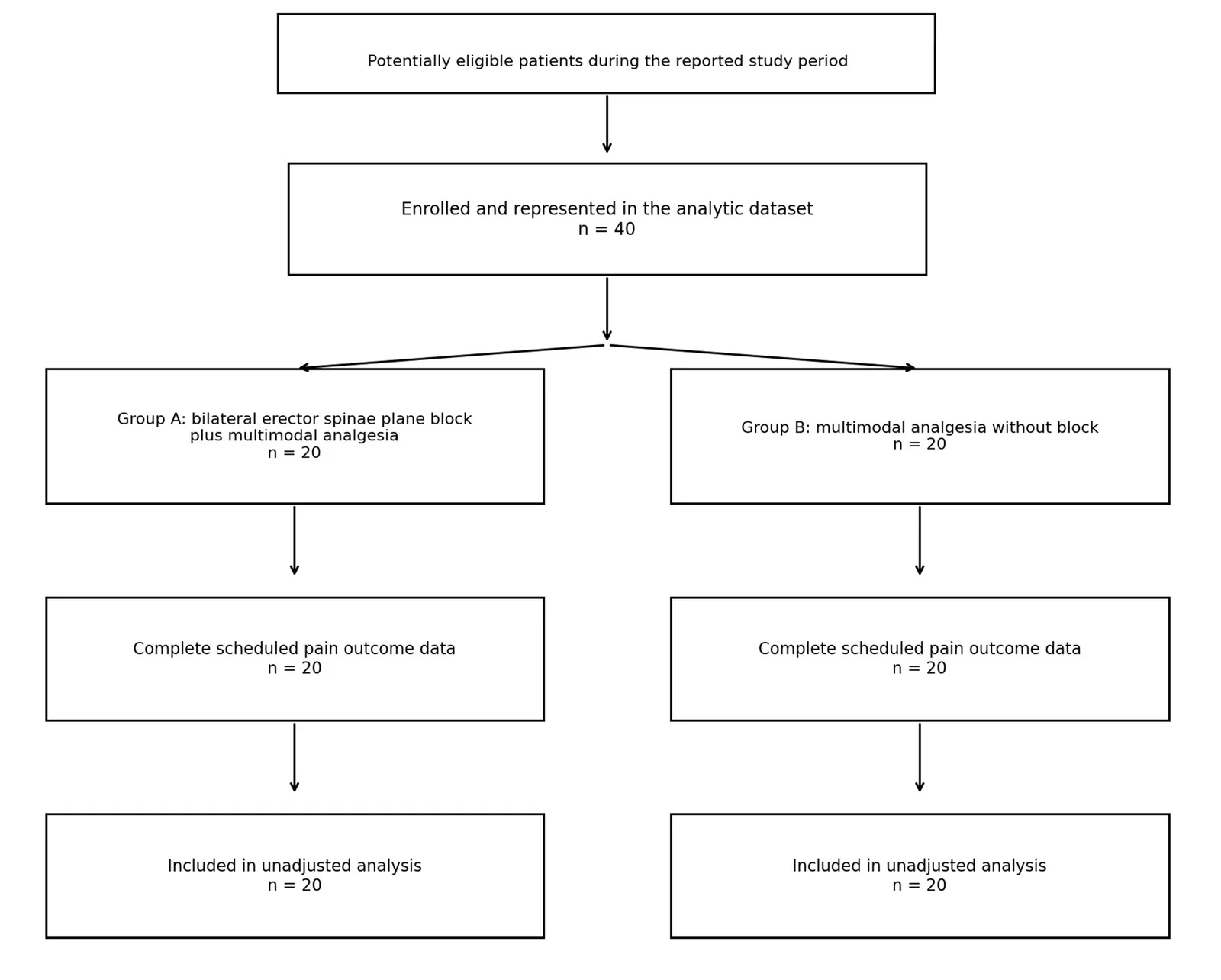
Participant flow diagram

Baseline age, weight, height, BMI, obesity category, and ASA physical status were similar between groups (Table 1). Sex was not included in the validated analytic dataset and was therefore not reported.

**Table 1:** Baseline characteristics of the analytic cohort Continuous-variable effect sizes are Cohen's d. Negative values indicate lower mean values in Group A. ASA: American Society of Anesthesiologists; BMI: body mass index; RR: risk ratio

Characteristic	Group A (block group) (n=20)	Group B (comparison group) (n=20)	Effect size	p value
Age (years), mean±SD	43.25 ± 11.89	43.45 ± 9.91	-0.02	0.954
Weight (kg), mean±SD	75.95 ± 11.29	77.90 ± 13.64	-0.16	0.625
Height (m), mean±SD	1.63 ± 0.09	1.64 ± 0.08	-0.13	0.681
BMI ( kg/m²), mean±SD	28.50 ± 2.63	28.93 ± 4.79	-0.11	0.727
Obesity (BMI ≥30 kg/m²), n (%)	6 (30.0)	8 (40.0)	RR 0.75 (0.32-1.77)	0.741
ASA physical status I, n (%)	1 (5.0%)	1 (5.0%)	Cramér V 0.00	1.000
ASA physical status II, n (%)	19 (95.0%)	19 (95.0%)		
ASA physical status III, n	0 (0.0%)	0 (0.0%)		

Postoperative pain

For resting pain, the crude main effect of group was significant, F(1,38)=23.21, p<0.001, partial eta squared=0.379. The Greenhouse-Geisser-corrected effect of time was significant, F(2.16,82.02)=4.36, p=0.014, partial eta squared=0.103, whereas the group-by-time interaction was not, F(2.16,82.02)=1.59, p=0.209, partial eta squared=0.040. Mauchly's W was 0.470 (p<0.001), and Greenhouse-Geisser epsilon was 0.719.

For movement pain, the group effect was significant, F(1,38)=31.56, p<0.001, partial eta squared=0.454, and pain declined over time, F(1,38)=25.26, p<0.001, partial eta squared=0.399. The group-by-time interaction was not significant, F(1,38)=0.67, p=0.418, partial eta squared=0.017. Pointwise comparisons are summarized in Table 2, and model-level effects are summarized in Table 3.

**Table 2:** Complete pointwise postoperative pain results. Negative mean differences and negative Hedges' g values favor Group A. The 0-hour comparison was not statistically significant. The two-hour parametric result was not confirmed by the Holm-adjusted Mann-Whitney sensitivity analysis (p=0.074). The 12- and 24-hour findings remained significant in sensitivity analyses.

Outcome/time	Group A, mean ± SD	Group B, mean ± SD	Mean difference A-B	95% CI	Holm p	Hedges' g
Rest, 0 hours	2.15 ± 1.14	2.80 ± 0.89	-0.65	-1.31 to 0.01	0.052	-0.62
Rest, 2 hours	2.20 ± 1.11	3.55 ± 2.14	-1.35	-2.45 to -0.25	0.036	-0.78
Rest, 12 hours	1.80 ± 0.70	3.40 ± 1.31	-1.60	-2.28 to -0.92	<0.001	-1.49
Rest, 24 hours	1.55 ± 0.60	2.55 ± 0.76	-1.00	-1.44 to -0.56	<0.001	-1.43
Movement, 12 hours	2.45 ± 1.00	3.95 ± 1.54	-1.50	-2.33 to -0.67	<0.001	-1.13
Movement, 24 hours	1.55 ± 0.60	2.70 ± 0.57	-1.15	-1.53 to -0.77	<0.001	-1.92

**Table 3:** Mixed repeated-measures model results.

Outcome	Effect	F	Degrees of freedom	p value	Partial eta squared
Resting pain	Group	23.21	1, 38	<0.001	0.379
Resting pain	Time, Greenhouse-Geisser	4.36	2.16, 82.02	0.014	0.103
Resting pain	Group × time, Greenhouse-Geisser	1.59	2.16, 82.02	0.209	0.040
Movement pain	Group	31.56	1, 38	<0.001	0.454
Movement pain	Time	25.26	1, 38	<0.001	0.399
Movement pain	Group × time	0.67	1, 38	0.418	0.017

Recorded rescue tramadol and postoperative nausea and vomiting

Recorded rescue tramadol use occurred in two (10.0%) participants in Group A and 14 (70.0%) participants in Group B. The risk ratio was 0.143 (95% CI 0.037-0.549), and the absolute risk difference was -0.60 (95% CI -0.84 to -0.36; p=0.00024). The descriptive number needed to treat to avoid one recorded rescue event was 1.67 (95% CI 1.19-2.78). Because the indication for every administration was not independently verifiable, these measures describe recorded use rather than confirmed protocol-triggered rescue.

Postoperative nausea and vomiting occurred in one participant (5.0%) in each group. The estimate was imprecise and does not establish comparative safety or equivalence. Binary postoperative outcomes are summarized in Table 4.

**Table 4:** Binary postoperative outcomes. NNT: number needed to treat

Outcome	Group A (n=20), n (%)	Group B (n=20), n (%)	Risk ratio (95% CI)	Risk difference (95% CI)	NNT (95% CI)	p value
Recorded rescue tramadol use	2 (10.0%)	14 (70.0%)	0.143 (0.037-0.549)	-0.60 (-0.84 to -0.36)	1.67 (1.19-2.78)	0.00024
Postoperative nausea and vomiting	1 (5.0%)	1 (5.0%)	1.00 (0.067-14.904)	0.00	Not applicable	1.000

## Discussion

The most clinically salient finding was the marked reduction in recorded rescue tramadol use among participants who received bilateral erector spinae plane block. The absolute difference was 60 percentage points, corresponding to a risk ratio of 0.143 and a descriptive number needed to treat of approximately two. This opioid-sparing signal is consistent with systematic reviews and controlled trials reporting reduced postoperative analgesic requirements after erector spinae plane block [6-17]. However, rescue analgesia was a secondary outcome, and the contemporaneous indication for every tramadol administration could not be verified. The finding should therefore be interpreted as a strong hypothesis-generating association rather than definitive evidence of protocol-adherent opioid sparing.

The prespecified primary endpoint also favored the block group: resting pain at 12 hours was 1.60 points lower. The standardized difference was large (Hedges' g=-1.49), but the absolute difference remained below the two-point threshold used for sample size planning. This distinction is important because statistical significance and standardized effect size do not establish that the prespecified absolute clinical target was reached. The absence of a significant group-by-time interaction further indicates that the data support an overall between-group difference rather than demonstrably different temporal trajectories.

The observed pattern is biologically plausible. Fascial plane spread may influence thoracic dorsal and ventral rami and provide multisegmental somatic and visceral analgesia [4,5]. Recent meta-analyses have generally found lower pain scores and opioid consumption after erector spinae plane block for laparoscopic cholecystectomy [6-8,17]. Nevertheless, heterogeneity in block level, timing, volume, concentration, comparator regimens, and outcome measurement limits direct comparison across studies.

This study has several limitations. The original group-allocation mechanism, assessor blinding, and complete pre-enrollment screening flow could not be independently verified, creating substantial potential for selection and detection bias. The sample was small, and no multivariable adjustment was performed; residual confounding therefore remains possible. The validated analytic dataset did not include sex, and unsupported sex counts were not reported. Technical details of the ultrasound system, transducer, needle, trajectory, and block-success criteria were not recorded, which limits procedural reproducibility. The rescue-tramadol analysis reflects recorded administrations rather than uniformly confirmed Numeric Rating Scale-triggered events. Finally, surveillance for local anesthetic systemic toxicity and other regional anesthesia complications [19-21] was not protocolized, so comparative safety cannot be established.

The results support further prospective evaluation using large cohort sizes, a documented allocation process, a complete screening log, blinded outcome assessment when feasible, standardized rescue criteria, complete block documentation, and prespecified safety surveillance. Future studies should preserve both absolute pain differences and opioid-use outcomes, because the present data suggest that the clinically meaningful signal may lie more in reduced rescue analgesia than in attainment of a two-point pain-score difference.

## Conclusions

In this study, bilateral erector spinae plane block was associated with lower unadjusted postoperative pain scores and substantially less recorded rescue tramadol use. The primary 12-hour resting-pain difference was statistically significant but remained below the prespecified two-point planning threshold. The reduction in recorded rescue analgesia was the most clinically salient finding, although incomplete verification of the indication for each administration limits causal interpretation. These results warrant confirmation in larger prospectively registered studies with documented allocation, complete participant flow, standardized rescue criteria, and structured safety surveillance.
